# Lateral gene transfer, rearrangement, reconciliation

**DOI:** 10.1186/1471-2105-14-S15-S4

**Published:** 2013-10-15

**Authors:** Murray Patterson, Gergely Szöllősi, Vincent Daubin, Eric Tannier

**Affiliations:** 1 INRIA Rhône-Alpes, 655 avenue de l'Europe, F-38344 Montbonnot, France; 2 Laboratoire de Biométrie et Biologie Évolutive, CNRS and Université de Lyon 1, 43 boulevard du 11 novembre 1918, F-69622 Villeurbanne, France; 3 Centrum Wiskunde & Informatica, Science Park 123, 1098 XG, Amsterdam, The Netherlands; 4 ELTE-MTA "Lendület" Biophysics Research Group 1117 Bp., Pázmány P. stny. 1A., Budapest, Hungary

## Abstract

**Background:**

Models of ancestral gene order reconstruction have progressively integrated different evolutionary patterns and processes such as unequal gene content, gene duplications, and implicitly sequence evolution via reconciled gene trees. These models have so far ignored lateral gene transfer, even though in unicellular organisms it can have an important confounding effect, and can be a rich source of information on the function of genes through the detection of transfers of clusters of genes.

**Result:**

We report an algorithm together with its implementation, DeCoLT, that reconstructs ancestral genome organization based on reconciled gene trees which summarize information on sequence evolution, gene origination, duplication, loss, and lateral transfer. DeCoLT optimizes in polynomial time on the number of rearrangements, computed as the number of gains and breakages of adjacencies between pairs of genes. We apply DeCoLT to 1099 gene families from 36 cyanobacteria genomes.

**Conclusion:**

DeCoLT is able to reconstruct adjacencies in 35 ancestral bacterial genomes with a thousand gene families in a few hours, and detects clusters of co-transferred genes. DeCoLT may also be used with any relationship between genes instead of adjacencies, to reconstruct ancestral interactions, functions or complexes.

**Availability:**

http://pbil.univ-lyon1.fr/software/DeCoLT/

## Introduction

The evolution of genomes can be explored at two different scales. At the chromosome level, rearrangements have been studied from the 1930's [1,2], and have progressively incorporated the possibility of unequal gene content, gene duplications and gene losses [3,4]. Later, but largely independently, in the 1960's, the evolution of genomic sequences began to be modeled [5,6], and has more recently been extended to include the duplication, loss and the lateral transfer of genes via the reconciliation of gene trees with a species tree [7-11]. These two scales have only met on a few occasions through the integration of phylogenies and rearrangements [12,13], using reconciled phylogenies to account for the duplication and loss of genes. Here, we build on these ideas and reconstruct ancestral gene order based on reconciled phylogenies that account for gene origination, duplication, loss, and transfer.

We propose an algorithm to simultaneously reconstruct all gene organizations along a species phylogeny, minimizing the number of gains and breakages of *adjacencies *that link consecutive genes on chromosomes. We build upon the dynamic programming principle proposed by Bérard *et al. *[13] and extend it to consider as input reconciliations containing lateral gene transfer produced by Szöllősi *et al. *[14].

We implement our algorithm naming the resulting software *DeCoLT*, in reference to DeCo [13] (Detection of Coevolution) with Lateral Transfers. We examine two datasets of gene trees from a single set of cyanobacteria species. The first set of gene trees is computed from sequence alignements only [15], and the second one is computed by a species tree aware method [16].

Our method and the efficiency of the computation is based on the hypothesis that adjacencies evolve independently from each other. While extant genomes consist either of a single or a relatively small number of linear or circular chromosomes, this hypothesis implies that reconstructed ancestral genomes may in theory exhibit more complex arrangements. For example an ancestral gene may be involved in more than two adjacencies, or a large number may have only a single adjacent gene. In the cyanobacteria dataset, extant genomes are all circular, and the ancestral genomes inferred by DeCoLT are also close to being circular with only a few deviations. Most deviations result from the absence of signal to reconstruct genomes in deep ancestors, but some are caused by errors in gene trees, leading to errors in ancestral gene contents. We observe that ancestral genome organizations computed from gene trees that are based on both the species tree and the sequence are closer to being circular than those computed from gene trees based on sequence alone. This argues for the validity of the reconstruction principle we present here and confirms that species tree aware methods produce more accurate gene trees.

## Context

**Figure 1:**
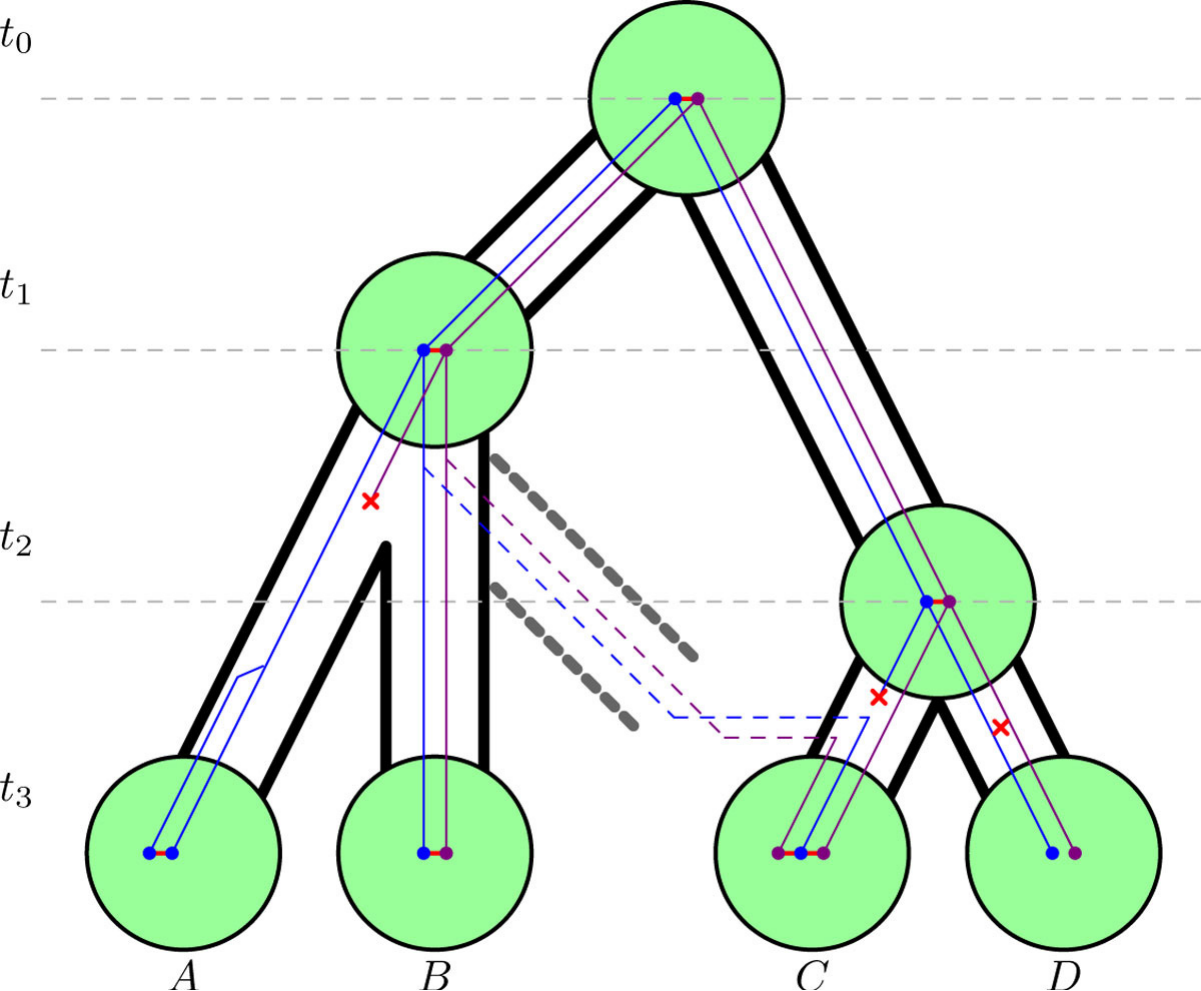
**Adjacency evolution**. The evolution of an adjacency within a dated species tree, along reconciled gene phylogenies. The gene trees are blue and purple, while red horizontal edges are adjacencies. The time slices *t*_0_, ..., *t*_3 _indicate in which order the speciation nodes (big green nodes) occur, and are used to localize genes in the species tree (a branch and a time slice give the coordinates of a gene or an event). Red crosses mean gene losses, for example in the branches leading to ***A ***or ***C ***(adjacencies are lost when one extremity is lost), or an adjacency breakage, for example in the branch leading to ***D ***(gene loss and adjacency breakages are different events, since a gene loss is not a rearrangement while a breakage is, and only rearrangements are counted in the objective function). Here one adjacency is gained in the branch leading to species ***C***, one is broken in the branch leading to species ***D***, and one is transferred from the branch leading to ***B ***to the branch leading to ***C***. The transfer implies first a speciation outside the species phylogeny, and then a transfer which can be in another time slice. A tandem duplication in the branch leading to ***A ***gives a new adjacency between the two copies.

A *dated species tree *is a rooted binary tree whose leaves are the *extant genomes *and internal nodes, the *ancestral genomes*, are totally ordered. This total order, which gives a relative dating to all internal nodes, is supposed to be known. It is, for example, constructed from branch length by a molecular clock technique or from informations on transfers [11]. The time interval between two consecutive internal nodes of a species tree in this total order (one is not necessarily the descendant of the other) defines a *time slice*. See Figure 1 for an example, where branches leading to *A*, *B *or the ancestor of *C *and *D *overlap two time slices while the others overlap one. Genomes contain a set of *genes *and a set of *adjacencies*, which are pairs of genes, the genes being the two *extremities *of the adjacency. An adjacency between two genes *a *and *b *is noted *ab *(*a *and *b *always are in the same species, and can be homologous or not). In extant genomes, an adjacency means that two genes are immediately consecutive, with no other gene between them on the chromosome (regardless of their physical distance), so most genes belong to exactly two adjacencies.

Genes of all genomes are partitioned into *homologous families*, and each family is organized in a *gene tree*, which is a rooted tree whose nodes are the genes, describing the pattern of descent within a family. Gene trees are *reconciled *with the species tree, which means that nodes and branches of gene trees are annotated to account for the particular history of the gene family. Possible events are origination (of the gene in the species tree), speciation (genes follow the species diversification), duplication, transfer, or loss. Transfers are the acquisition of a gene by a genome in the species tree from a genome outside the species tree. Indeed genes at the origin of transfers almost always belong to unsampled or extinct species [14]. That is why speciation does not only happen at the nodes of the species tree. A gene can also leave the species tree by speciation from a species tree branch, and be transferred back later (see Figure 1 for an example). But we assume that genes do not diversify (either by speciation or by duplication) outside the species tree. Every event on a gene tree is associated to a branch and a time slice of the species tree.

The input to our method is a dated species tree, a set of reconciled gene trees and the set of extant adjacencies. Reconciled gene trees yield ancestral genes (dots inside green circles in Figure 1). The problem will be to construct the ancestral adjacencies, given this input. In practice the input is provided by methods and software described in Szöllősi *et al*.'s trilogy [11,14,16]. The first paper of this trilogy explains how to find the dated species tree, the second one how to reconcile gene trees taking extinct or unsampled species into account, and the third one how to reconstruct species tree aware gene trees.

We construct ancestral adjacencies in a manner that minimizes the number of rearrangements along the species phylogeny. This number is computed as the number of gains and breakages of adjacencies necessary to explain all extant adjacencies. For example, if we infer no ancestral adjacencies at all, then the value of this objective function is proportional to the number of extant adjacencies, because all of them are gained independently. If we propose an adjacency in an ancestral genome which is a common ancestor of a set of adjacencies, then the value decreases because all adjacencies in that set are explained by a unique gain (see Figure 1, where three extant adjacencies can be explained with two gains and a breakage).

We then have to describe how an ancestral adjacency *propagates *within reconciled gene trees so that it can be recognized as an ancestor of an extant one.

## Propagation rules

**Figure 2:**
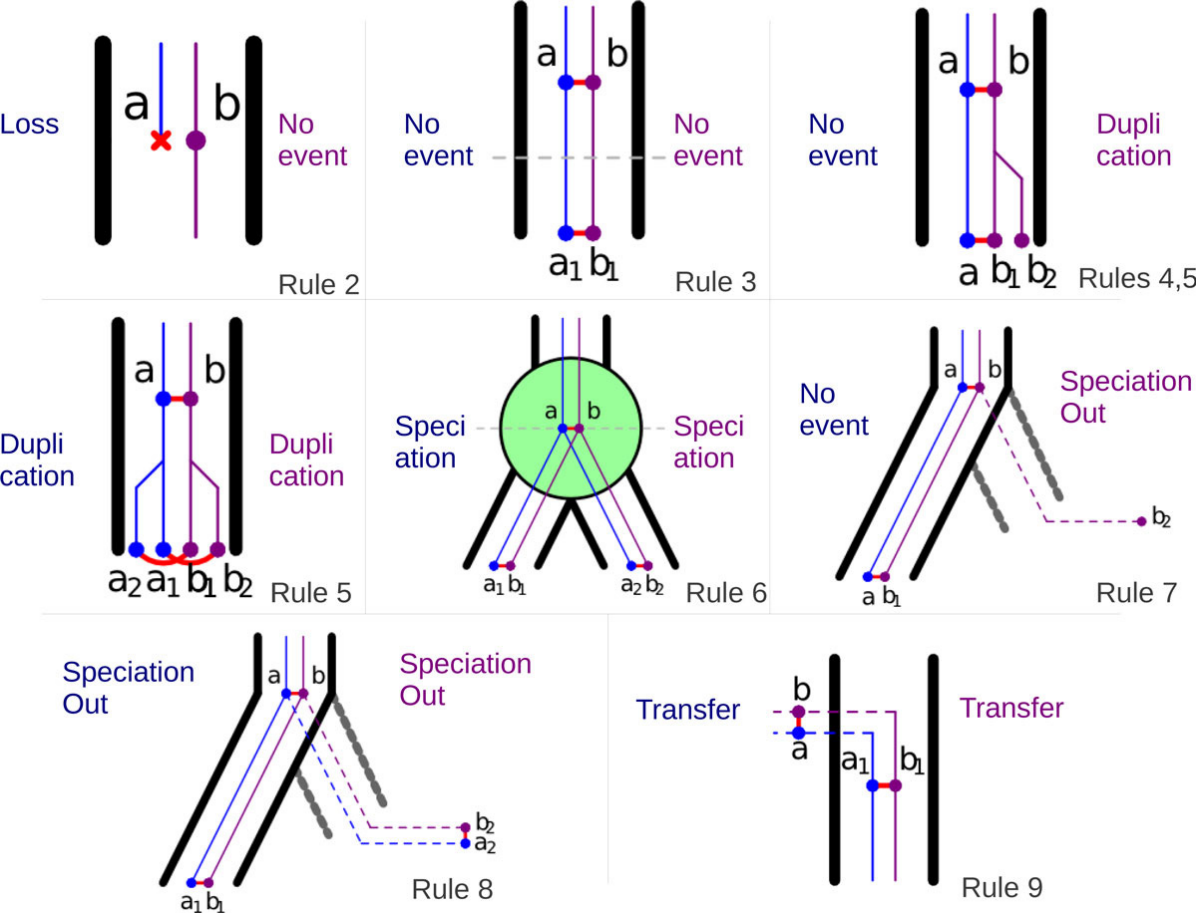
**Propagation rules**. Propagation rules for an adjacency *ab*: a function of the events happening to its extremities *a *and *b *(events happening to *a *are written on the left, events happening to *b *are written on the right). If *a*_1_, *a*_2_, *b*_1 _and *b*_2 _are the children of *a *and *b*. Numbers on each subsquare are recurrence rules following the propagation rule. Recurrence rules add the possibility of rearrangements at each step. For combination of duplication or loss with another event not mentioned here, follow the rule with "no event" (the duplication or loss is supposed to happen first).

The two extremities of an adjacency are clearly always in the same species (extant or ancestral) at the same time slice. If there is an adjacency between two ancestral genes *a *and *b*, it is propagated to the descendants, in the absence of rearrangements, following the rules described in Figure 2, according to the events happening to genes *a *and *b*.

A *history *is a set of ancestral and extant adjacencies. In a history, any adjacency which does not have a parent identified by the propagation rules yields an adjacency *gain*. A *breakage *is inferred when an adjacency is present in the history but one of its descendants according to the propagation rules is not. The *cost *of a history is the number of gains and breakages it yields.

## Algorithm

We compute a minimum cost history by writing a dynamic programming algorithm following the propagation rules and adding adjacency gains and breakages with costs that are considered in the optimization. In order to solve a more general problem and to present the recurrence formulas more clearly, gains and breakages are assigned a cost, which could be different, and we minimize on the number of events weighted by their cost. In practice we always use the algorithm with equal costs, thus minimizing the sum of the number of gains and breakages.

### Classes of adjacencies

Two adjacencies are *homologous *with respect to a particular history if they descend from a common ancestor following the propagation rules. Homology of adjacencies is an equivalence relation. We first state a necessary condition for a set of adjacencies to be homologous in order to restrict the search space for homology.

Two extant adjacencies *a*_1_*b*_1 _and *a*_2_*b*_2 _are *possibly homologous *if there are two ancestral genes *a *and *b *of an ancestral genome *G*, such that *a *(resp., *b*) is an ancestor of *a*_1 _and *a*_2 _(resp., *b*_1 _and *b*_2_). This simply tells us that in order to find a common ancestor of two adjacencies, there has to exist two genes being the extremities of this adjacency. So if two adjacencies are homologous with respect to a particular history then they are possibly homologous (the definition of possible homology is independent from any history). Possible homology, defined on two adjacencies, is obviously a symmetric and reflexive relation. It is also transitive, partitioning the set of extant adjacencies into equivalence classes.

Consequently, homology can be searched within a class. For each class {*a*_1_*b*_1_, ..., *a_k _b_k_*}, there are two genes *a *and *b*, such that *a *(resp. *b*) is an ancestor of all *a_i _*(resp., *b_i_*). Among the possible such genes *a *and *b *for a class, we call the highest distinct ones the *roots *of that class. We then work with the disjoint subtrees rooted by *a *and *b*, and find a history following the propagation rules for all adjacencies whose extremities are descendants of *a *and *b*. Hence, it is sufficient to search within pairs of trees to construct a history.

### Recurrence formulas within one class

For any two gene tree nodes *a *and *b*, for which *s*(*a*) = *s*(*b*), let *c*_1_(*a, b*) be the minimum cost of a history for the two gene subtrees rooted at *a *and *b*, assuming there is an adjacency between *a *and *b*, and let *c*_0_(*a, b*) be the minimum cost of a history for two gene subtrees rooted at *a *and *b*, assuming there is no adjacency between *a *and *b*. The values of *c*_1_(*a, b*) and *c*_0_(*a, b*) are recursively computed, according to the events annotating *a *and *b*.

The principle of these formulas is to describe the propagation rules and add to them the possibility of rearrangements (gains and breakages of adjacencies). We compute *c*_1_(*a, b*) and *c*_0_(*a, b*) as a function of *c*_1 _and *c*_0 _for the children of *a *and *b*. So we have to consider all combinations of presence or absence of adjacencies between the children. That is why some cases may imply up to 16 different subcases because of the symmetry of the children of *a *and *b*, if they are in the same species.

Given a node *u *(*u *= *a *or *u *= *b*), *u*_1 _is the first (or only child of *u *in the case that *u *has only one child), while *u*_2 _is the second child. We write *E*(*u*) to denote the event at node *u*, where *E*(*u*) = *Extant *when *u *is a leaf of a gene tree corresponding to an extant gene.

**Case 1 ***E*(*a*) = *Extant *and *E*(*b*) = *Extant (both nodes are leaves)*.

In this case, if *ab *is an adjacency then *c*_1_(*a, b*) = 0 and *c*_0_(*a, b*) = ∞, else *c*_1_(*a, b*) = ∞ and *c*_0_(*a, b*) = 0.

**Case 2 ***E*(*a*) = *GeneLoss (one of the genes is lost, any event may happen to the other)*.

In this case *c*_1_(*a, b*) = 0 and *c*_0_(*a, b*) = 0.

**Case 3 ***E*(*a*) = *NoEvent *and *E*(*b*) = *NoEvent (both gene trees are changing time slice without any event)*.

In this case *c*_1_(*a, b*) = min{*c*_1_(*a*_1_, *b*_1_), *c*_0_(*a*_1_, *b*_1_) + *C*(*Break*)} and *c*_0_(*a, b*) = min{*c*_0_(*a*_1_, *b*_1_), *c*_1_(*a*_1_, *b*_1_) + *C*(*Gain*)}.

**Case 4 ***E*(*a*) ∈ {*Extant, NoEvent, Speciation, SpeciationOut*} and *E*(*b*) = *GeneDuplication*

In this case we suppose that the duplication of *b *happens before any event in the gene tree containing *a*. Here, *c*_1_(*a, b*) = **D1**, and *c*_0_(*a, b*) = **D0**, where

$$D1=\text{min}\left\{\begin{array}{l} c_{1}\left(a,b_{1}\right)+c_{0}\left(a,b_{2}\right),\\ c_{0}\left(a,b_{1}\right)+c_{1}\left(a,b_{2}\right),\\ c_{1}\left(a,b_{1}\right)+c_{1}\left(a,b_{2}\right)+C\left(Gain\right),\\ c_{0}\left(a,b_{1}\right)+c_{0}\left(a,b_{2}\right)+C\left(Break\right) \end{array}\right)\;D0=\text{min}\left\{\begin{array}{l} c_{0}\left(a,b_{1}\right)+c_{0}\left(a,b_{2}\right),\\ c_{0}\left(a,b_{1}\right)+c_{1}\left(a,b_{2}\right)+C\left(Gain\right),\\ c_{1}\left(a,b_{1}\right)+c_{0}\left(a,b_{2}\right)+C\left(Gain\right),\\ c_{1}\left(a,b_{1}\right)+c_{1}\left(a,b_{2}\right)+2*C\left(Gain\right) \end{array}\right)$$

**Case 5 ***E*(*a*) = *GeneDuplication *and *E*(*b*) = *GeneDuplication*.

In this case *c*_1_(*a, b*) = min(**D1**, **D12**, **D12**) where

**D1 **(defined in Case 4) is the cost in the case where the *a *duplication comes first,

**D2 **is the cost in the case where the *a *duplication comes first, and

$$D2=\text{min}\left\{\begin{array}{l} c_{1}\left(a_{1},b\right)+c_{0}\left(a_{2},b\right),\\ c_{0}\left(a_{1},b\right)+c_{1}\left(a_{2},b\right),\\ c_{1}\left(a_{1},b\right)+c_{1}\left(a_{2},b\right)+C\left(Gain\right),\\ c_{0}\left(a_{1},b\right)+c_{0}\left(a_{2},b\right)+C\left(Break\right) \end{array}\right)$$

**D12 **is the cost in the case where the *a *and *b *duplications are simultaneous, where **D12 **= min (over all 16 of the following cases):

$$\left\{\begin{array}{l} \left(1\right)\;c_{1}\left(a_{1},b_{1}\right)+c_{1}\left(a_{2},b_{2}\right)+c_{0}\left(a_{1},b_{2}\right)+c_{0}\left(a_{2},b_{1}\right),\\ \left(2\right)\;c_{1}\left(a_{1},b_{1}\right)+c_{1}\left(a_{2},b_{2}\right)+c_{0}\left(a_{1},b_{2}\right)+c_{1}\left(a_{2},b_{1}\right)+C\left(Gain\right),\\ \left(3\right)\;c_{1}\left(a_{1},b_{1}\right)+c_{1}\left(a_{2},b_{2}\right)+c_{1}\left(a_{1},b_{2}\right)+c_{0}\left(a_{2},b_{1}\right)+C\left(Gain\right),\\ \left(4\right)\;c_{1}\left(a_{1},b_{1}\right)+c_{1}\left(a_{2},b_{2}\right)+c_{1}\left(a_{1},b_{2}\right)+c_{1}\left(a_{2},b_{1}\right)+2*C\left(Gain\right),\\ \left(5\right)\;c_{1}\left(a_{1},b_{1}\right)+c_{0}\left(a_{2},b_{2}\right)+c_{0}\left(a_{1},b_{2}\right)+c_{0}\left(a_{2},b_{1}\right)+C\left(Break\right),\\ \left(6\right)\;c_{1}\left(a_{1},b_{1}\right)+c_{0}\left(a_{2},b_{2}\right)+c_{0}\left(a_{1},b_{2}\right)+c_{1}\left(a_{2},b_{1}\right)+C\left(Gain\right)+C\left(Break\right),\\ \left(7\right)\;c_{1}\left(a_{1},b_{1}\right)+c_{0}\left(a_{2},b_{2}\right)+c_{1}\left(a_{1},b_{2}\right)+c_{0}\left(a_{2},b_{1}\right)+C\left(Gain\right)+C\left(Break\right),\\ \left(8\right)\;c_{0}\left(a_{1},b_{1}\right)+c_{1}\left(a_{2},b_{2}\right)+c_{0}\left(a_{1},b_{2}\right)+c_{0}\left(a_{2},b_{1}\right)+C\left(Break\right),\\ \left(9\right)\;c_{0}\left(a_{1},b_{1}\right)+c_{1}\left(a_{2},b_{2}\right)+c_{0}\left(a_{1},b_{2}\right)+c_{1}\left(a_{2},b_{1}\right)+C\left(Gain\right)+C\left(Break\right),\\ \left(10\right)\;\;c_{0}\left(a_{1},b_{1}\right)+c_{1}\left(a_{2},b_{2}\right)+c_{1}\left(a_{1},b_{2}\right)+c_{0}\left(a_{2},b_{1}\right)+C\left(Gain\right)+C\left(Break\right),\\ \left(11\right)\;\;c_{0}\left(a_{1},b_{1}\right)+c_{0}\left(a_{2},b_{2}\right)+c_{1}\left(a_{1},b_{2}\right)+c_{1}\left(a_{2},b_{1}\right),\\ \left(12\right)\;\;c_{0}\left(a_{1},b_{1}\right)+c_{1}\left(a_{2},b_{2}\right)+c_{1}\left(a_{1},b_{2}\right)+c_{1}\left(a_{2},b_{1}\right)+C\left(Gain\right),\\ \left(13\right)\;\;c_{1}\left(a_{1},b_{1}\right)+c_{0}\left(a_{2},b_{2}\right)+c_{1}\left(a_{1},b_{2}\right)+c_{1}\left(a_{2},b_{1}\right)+C\left(Gain\right),\\ \left(14\right)\;\;c_{0}\left(a_{1},b_{1}\right)+c_{0}\left(a_{2},b_{2}\right)+c_{1}\left(a_{1},b_{2}\right)+c_{0}\left(a_{2},b_{1}\right)+C\left(Break\right),\\ \left(15\right)\;\;c_{0}\left(a_{1},b_{1}\right)+c_{0}\left(a_{2},b_{2}\right)+c_{0}\left(a_{1},b_{2}\right)+c_{1}\left(a_{2},b_{1}\right)+C\left(Break\right),\\ \left(16\right)\;\;c_{0}\left(a_{1},b_{1}\right)+c_{0}\left(a_{2},b_{2}\right)+c_{0}\left(a_{1},b_{2}\right)+c_{0}\left(a_{2},b_{1}\right)+2*C\left(Break\right) \end{array}\right)$$

The multiplication of the cases come from the symmetry of the children of both *a *and *b *(for example, (1) is the symmetric subcase of (11)). As there are four children, there are four possible pairings between children from *a *and *b*, so we examine all combinations, that is, 2^4 ^= 16.

Here, *c*_0_(*a, b*) = **D00**, where

$$D00=\text{min}\left\{\begin{array}{l} D0\\ c_{0}\left(a_{1},b\right)+c_{0}\left(a_{2},b\right),\\ c_{0}\left(a_{1},b\right)+c_{1}\left(a_{2},b\right)+C\left(Gain\right),\\ c_{0}\left(a_{1},b\right)+c_{0}\left(a_{2},b\right)+C\left(Gain\right),\\ c_{1}\left(a_{1},b\right)+c_{1}\left(a_{2},b\right)+2*C\left(Gain\right) \end{array}\right)$$

**Case 6 ***E*(*a*) = *Speciation *and *E*(*b*) = *Speciation*.

We assume without loss of generality that *s*(*a*_1_) = *s*(*b*_1_) and *s*(*a*_2_) = *s*(*b*_2_). Here, *c*_1_(*a, b*) = **S1 **and *c*_0_(*a, b*) = **S0**, where

$$S1=\text{min}\left\{\begin{array}{l} c_{1}\left(a_{1},b_{1}\right)+c_{1}\left(a_{2},b_{2}\right),\\ c_{1}\left(a_{1},b_{1}\right)+c_{0}\left(a_{2},b_{2}\right)+C\left(Break\right),\\ c_{0}\left(a_{1},b_{1}\right)+c_{1}\left(a_{2},b_{2}\right)+C\left(Break\right),\\ c_{0}\left(a_{1},b_{1}\right)+c_{0}\left(a_{2},b_{2}\right)+2*C\left(Break\right) \end{array}\right)\;S0=\text{min}\left\{\begin{array}{l} c_{0}\left(a_{1},b_{1}\right)+c_{0}\left(a_{2},b_{2}\right),\\ c_{1}\left(a_{1},b_{1}\right)+c_{0}\left(a_{2},b_{2}\right)+C\left(Gain\right),\\ c_{0}\left(a_{1},b_{1}\right)+c_{1}\left(a_{2},b_{2}\right)+C\left(Gain\right),\\ c_{1}\left(a_{1},b_{1}\right)+c_{1}\left(a_{2},b_{2}\right)+2*C\left(Gain\right) \end{array}\right)$$

**Case 7 ***E*(*a*) ∈ {*Extant, NoEvent, Speciation*} and *E*(*b*) = *SpeciationOut*.

In this case *c*_1_(*a, b*) = *c*_1_(*a, b*_1_) and *c*_0_(*a, b*) = *c*_0_(*a, b*_1_).

**Case 8 ***E*(*a*) = *SpeciationOut *and *E*(*b*) = *SpeciationOut*.

We assume without loss of generality that *a*_1 _(resp., *b*_1_) is the child that remains inside the species tree, while *a*_2 _(resp., *b*_2_) is the child that leaves the tree. In this case, *c*_1_(*a, b*) = *c*_1_(*a*_1_, *b*_1_) + min{*c*_1_(*a*_2_, *b*_2_), *c*_0_(*a*_2_, *b*_2_) + *C*(*Break*)} and *c*_0_(*a, b*) = *c*_0_(*a*_1_, *b*_1_) + min{*c*_0_(*a*_2_, *b*_2_), *c*_1_(*a*_2_, *b*_2_) + *C*(*Gain*)}.

**Case 9 ***E*(*a*) = *Transfer *and *E*(*b*) = *Transfer*.

In this case, *c*_1_(*a, b*) = min{*c*_1_(*a*_1_, *b*_1_), *c*_0_(*a*_1_, *b*_1_) + *C*(*Break*)} and *c*_0_(*a, b*) = min{*c*_0_(*a*_1_, *b*_1_), *c*_1_(*a*_1_, *b*_1_) + *C*(*Gain*)}.

Observe that if *E*(*a*) = *Transfer *and *E*(*b*) ∉  {*Transfer, GeneLoss*}, or if *a *and *b *do not have a SpeciationOut ancestor in the same species, then (*a, b*) will form the root of an additional equivalence class. Indeed in this case *c*_1_(*a, b*) and *c*_0_(*a, b*) cannot be recursively called.

### Backtracking Procedure

First, the dynamic programming matrix *M *[*a, b*] containing a cell for each pair (*a, b*) of nodes in the respective gene trees is created by following the recurrence rules for each equivalence class.

Then, after applying the classical backtracking procedure on *M*, the optimal (minimum cost) history is then obtained by choosing the minimum among *c*_1 _+ *C*(*Gain*) and *c*_0 _for the roots of all classes.

### Complexity

Let *m *be the number of gene trees, *n *be the maximum number of genes in a gene tree, and *s *be the number of species. There are *s - *1 time slices, so every branch of a gene tree may be subdivided as many as *s - *1 ≤ *n *times, which gives at most *n*^2 ^events (most of them are *NoEvent *events) in a given tree. The number of equivalence classes is *O*(*m*^2^), and hence there are *O*(*m*^2^*n*^4^) comparisons computed during the initial construction of dynamic programming matrix *M*.

In practice, the number of equivalence classes is much smaller, closer to *m *than *O*(*m*^2^), and the majority of the *O*(*n*^2^) events each tree are *NoEvent *events. On the cyanobacteria dataset of (*m *=) 1099 families from (*n *=) 36 genomes, our implementation, DeCoLT, of this algorithm constructed the adjacencies in under 3 hours on a standard desktop computer.

## Cyanobacteria ancestral genomes

**Figure 3:**
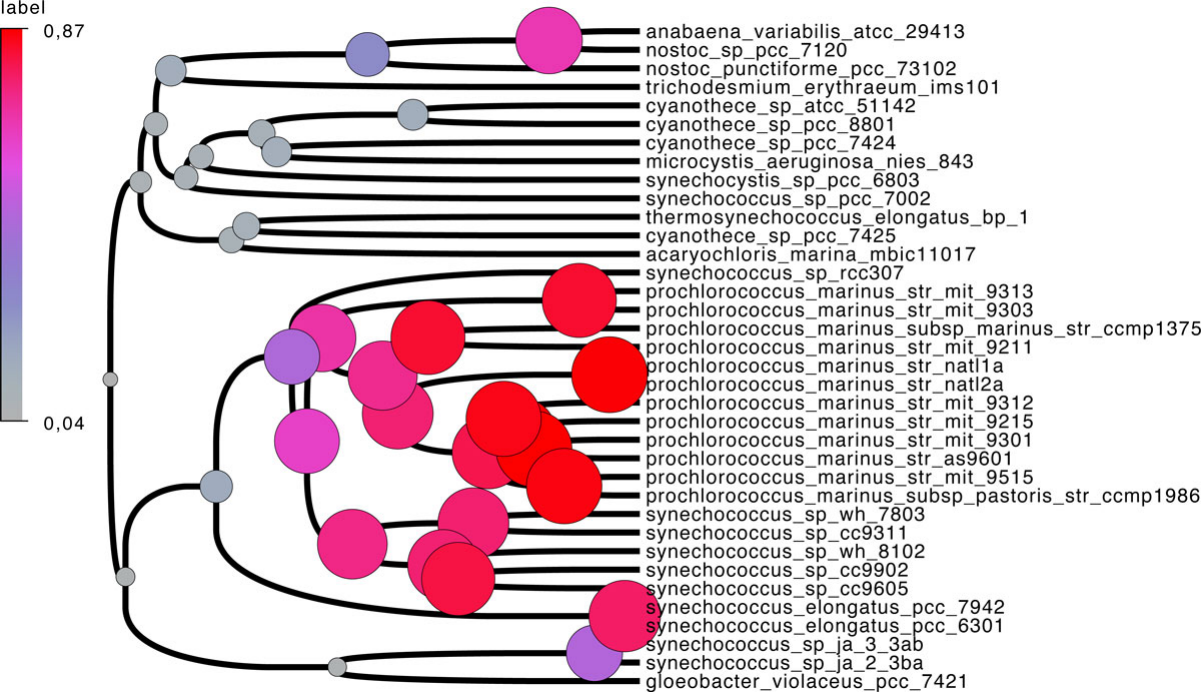
**Cyanobacteria phylogeny**. Cyanobacteria dated species tree. The size (area) and colours of internal nodes is the ratio of the number of adjacencies over the number of genes in every ancestral species.

The algorithm has been implemented and run on two datasets. They both have the same species tree (depicted in Figure 3), on the same set of 36 extant genomes from cyanobacteria and the same extant adjacencies.

They differ by their set of gene trees. One of them is the *sequence trees*, which are maximum likelihood trees constructed from a model of sequence evolution using multiple alignments of protein sequences of extant genes from each family, taken from [11].

The other is the *ALE *trees, which are maximum likelihood trees constructed from a model of sequence evolution in conjunction with a birth and death branching model to account for origination, duplication, transfer and loss, taken from [16]. As transfers are very likely to involve lineages outside any given phylogeny [14], reconciled trees have nodes leaving the species tree (*SpeciationOut*) and nodes transferring to the species tree (*Transfer*). Note that the reconciliations may contain "speciation outside" nodes, which mean diversification of a gene outside the species tree, and our algorithm do not handle these nodes for the moment. In order to handle these datasets we simply removed these nodes, which has the effect of cutting some trees into pieces.

**Figure 4:**
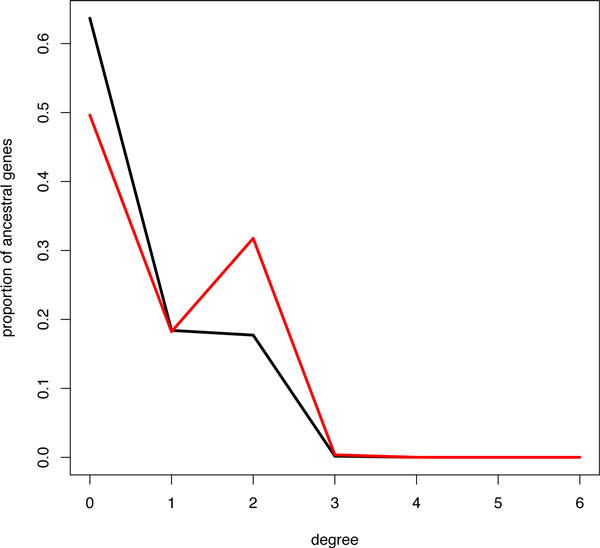
**Circularity of ancestral genomes**. On the *x *axis is the degree of a gene, that is, the number of adjacencies it belongs to, and on the *y *axis there is the proportion of genes with this degree. In black, there are the values for the sequence trees and in red for ALE trees.

For both datasets, ancestral adjacencies were computed using DeCoLT. The degree of each ancestral gene (the number of adjacencies it belongs to) was computed, and we then plotted the proportion of ancestral genes having degree *k *for *k *between 0 and 6 (Figure 4).

There are almost no genes with degree larger than 2 in either dataset. The proportion of genes with degree 2 increases from 17% for sequence trees to 31% for ALE trees. This means that we: (i) accurately reconstruct ancestral adjacencies because they all have a circular structure; and (ii) the quality of gene trees nearly doubles the resolution of ancestral genomes. Finally, having only 31% of ancestral genes with degree two means that a large part of the gene order signal is lost in this very deep branch. However, this is not the case for ancestral genomes. The size of the nodes on Figure 3 indicates the ratio between the number of adjacencies and the number of genes in each genome (the ideal ratio is 1). In the *Prochlorococcus *clade, over 80% of the genomes are reconstructed whereas it drops to nearly 0% in deeper nodes.

We found that 64 clusters of genes were co-transferred: transferred adjacencies were detected, as well as 28 clusters of co-duplicated genes during the evolution of cyanobacteria. Most are simply pairs of genes, but there is a cluster of 4 co-transferred genes, four clusters of 3 co-transferred genes, and two clusters of 3 co-duplicated genes.

## Discussion

The optimizing property of the algorithm follows from the exact translation of the propagation rules into the recurrence formulas, adding all possibilities of rearrangements in each step. It is a generalization of the reconstruction of discrete ancestral characters solved by Sankoff-Rousseau type algorithms [17]. To see this, one can observe that our framework is strictly equivalent to the Sankoff-Rousseau algorithm [17] in the case where there are no events in the trees.

Further improvements in the method would consist in adding the possibility of homolog replacement when a gene is transferred: for the moment any transfer yields rearrangements whereas some genes might replace an homologous one, keeping the gene order unchanged. We could also think of avoiding rearrangements caused by origination and losses of genes, which, for the moment, necessarily yield several adjacency gains and losses.

Future work will also consist in deriving function information from co-transfers, and trying the same principles on other kinds of relations than adjacencies, starting for example from the relation between domains forming the same gene.

## Competing interests

The authors declare that they have no competing interests.

## Authors' contributions

MP, GS, VD and ET devised the algorithm and wrote the paper. MP programmed the software.
